# 2-{3-Methyl-2-[(2*Z*)-pent-2-en-1-yl]cyclo­pent-2-en-1-yl­idene}-*N*-phenylhydrazinecarbo­thio­amide

**DOI:** 10.1107/S2414314623009719

**Published:** 2023-11-14

**Authors:** Adriano Bof Oliveira, Leandro Bresolin, Johannes Beck, Jörg Daniels

**Affiliations:** a Departamento de Química, Universidade Federal de Sergipe, Av. Marcelo Deda Chagas s/n, Campus Universitário, 49107-230 São Cristóvão-SE, Brazil; b Escola de Química e Alimentos, Universidade Federal do Rio Grande, Av. Itália km 08, Campus Carreiros, 96203-900 Rio Grande-RS, Brazil; c Institut für Anorganische Chemie, Rheinische Friedrich-Wilhelms-Universität Bonn, Gerhard-Domagk-Strasse 1, D-53121 Bonn, Germany; Goethe-Universität Frankfurt, Germany

**Keywords:** *cis*-jasmone, 4-phen­yl­thio­semi­carbazone, thio­semicarbazone, jasmone, crystal structure, Hirshfeld analysis.

## Abstract

The hydro­chloric acid-catalyzed equimolar reaction between *cis*-jasmone and 4-phenyl­thio­semicarbazide yielded the title compound, C_18_H_23_N_3_S (common name: *cis*-jasmone 4-phenyl­thio­semicarbazone). Concerning the hydrogen bonding, an N—H⋯N intra­molecular inter­action is observed, forming a ring with graph-set motif *S*(5). In the crystal, the mol­ecules are connected into centrosymmetric dimers by pairs of N—H⋯S and C—H⋯S inter­actions, forming rings of graph-set motifs *R*_2_^2^(8) and *R*_2_^1^(7), with the sulfur atoms acting as double acceptors. The thio­semicarbazone entity is approximately planar, with the maximum deviation from the mean plane through the N/N/C/S/N atoms being 0.0376 (9) Å (the r.m.s.d. amounts to 0.0234 Å). The mol­ecule is substantially twisted as indicated by the dihedral angle between the thio­semicarbazone fragment and the phenyl ring, which amounts to 56.1 (5)°, and because of the jasmone fragment, which bears a chain with *sp* ^3^-hybridized carbon atoms in the structure. The Hirshfeld surface analysis indicates that the major contributions for the crystal cohesion are: H⋯H (65.3%), H⋯C/C⋯H (16.2%), H⋯S/S⋯H (10.9%) and H⋯N/N⋯H (5.5%).

## Abstract 2

The synthesis, crystal structure and Hirshfeld analysis of the *cis*-jasmone 4-phenyl­thio­semicarbazone is reported. The mol­ecules are linked into centrosymmetric dimers *via* pairs of N—H⋯S and C—H⋯S inter­actions, with graph-set motifs 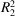 (8) and 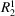 (7). In addition, one N—H⋯N intra­molecular inter­action is observed, with graph-set motif *S*(5). The crystal structure resembles a zigzag motif when viewed along [010].

## Graphical abstract

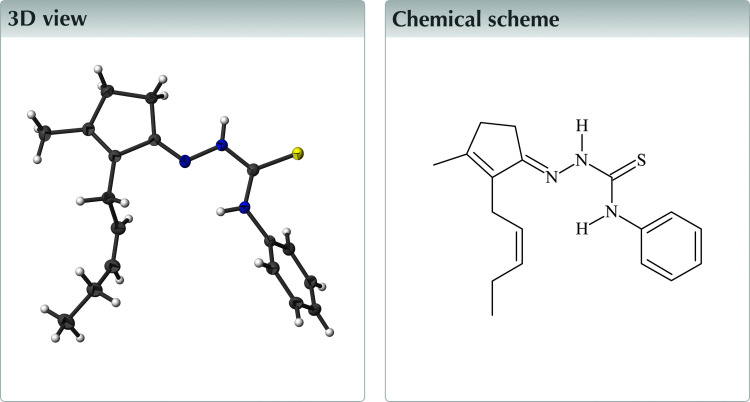

## Structure description

Thio­semicarbazone derivatives (**TSC**s), which are characterized by the [*R*_1_ *R*_2_C=N—N(H)—C(=S)—N*R*_3_ *R*_4_] functional group, were reported more than a century ago (Freund & Schander, 1902 ▸), while the synthesis of jasmone derivatives can be traced back to the early 1930s (Ruzicka & Pfeiffer, 1933 ▸). Concerning **TSC** chemistry, thio­semicarbazone mol­ecules are the major product of the reaction between thio­semi­carbazide derivatives [H_2_N—N(H)—C(=S)—N*R*_3_ *R*_4_] and aldehydes or ketones [*R*_1_ *R*_2_C=O]. Thio­semicarbazides have been employed as analytical reagents in organic chemistry for the detection of the [*R*_1_ *R*_2_C=O] functional group by a condensation reaction through nucleophilic attack of the [H_2_N—] thio­semicarbazide fragment on the carbonyl group. Thio­semicarbazone chemistry gained new perspectives in the mid-1940s when some derivatives were pointed out in *in vitro* essays to be tuberculostatic agents (Domagk *et al.*, 1946 ▸). From these early times, this chemistry evolved into a large class of compounds with a wide range of applications across several scientific disciplines. The facile experimental procedure for the synthesis, combined with the vast structural diversity of the starting materials, *i.e.*, aldehydes and ketones, lead to a large number of **TSC**s. As a result of their mol­ecular structure and the respective Lewis basicity (nitro­gen atoms, with some more *hard* character, and the *soft* sulfur atom), allowing for chemical bonding with different metal centers in diverse modes, *e.g.*, bridging, chelating or terminal, thio­semicarbazones found several applications in coordination chemistry. For the synergetic effect of thio­semicarbazones and metal centers, see: Lobana *et al.* (2009 ▸). For the application on diagnostic medical imaging of **TSC** complexes, see: Dilworth & Hueting (2012 ▸) and for the application of **TSC** coordination compounds on theranostics, see: Parrilha *et al.* (2022 ▸). For electrocatalytic hydrogen production using a Pd^II^ complex with the 4-{bis­[4-(*p*-meth­oxy­phen­yl)thio­semicarbazone]}-2,3-butane derivative, which is relevant for the energy research today, see: Straistari *et al.* (2018 ▸). For biological applications of **TSC**s and their complexes, see: Singh *et al.* (2023 ▸). For the anti­fungal activity and the crystal structure of the non-substituted *cis*-jasmone thio­semicarbazone, see: Orsoni *et al.*, (2020 ▸) and for another report concerning the fungistatic effect of this **TSC** derivative, see: Jamiołkowska *et al.* (2022 ▸). For the application of thio­semicarbazones complexes as single-mol­ecule precursors in the synthesis of nanostructured metal sulfides, see: Palve & Garje (2011 ▸) for ZnS, Pawar *et al.* (2016 ▸) for CdS and Pawar & Garje (2015 ▸) for CoS nanocrystalline materials. Regarding the use of a **TSC** on the formation of palladium nanoparticles for the Suzuki–Miyaura cross-coupling catalytic application, see: Kovala-Demertzi *et al.* (2008 ▸). Finally, to cite another example of their applications, thio­semicarbazones are employed as corrosion inhibitors. For an experimental and theoretical study regarding the corrosion-inhibitory property of **TSC**s applied for carbon steel AISI 1020 in a hydro­chloric acid medium, see: Goulart *et al.* (2013 ▸). For a theoretical approach of **TSC** dimers as corrosion inhibitors, see: Silva & Martínez-Huitle (2021 ▸).

As part of our inter­est in this chemistry, we report herein the synthesis, crystal structure and Hirshfeld analysis of the *cis*-jasmone 4-phenyl­thio­semicarbazone.

**Figure 1:**
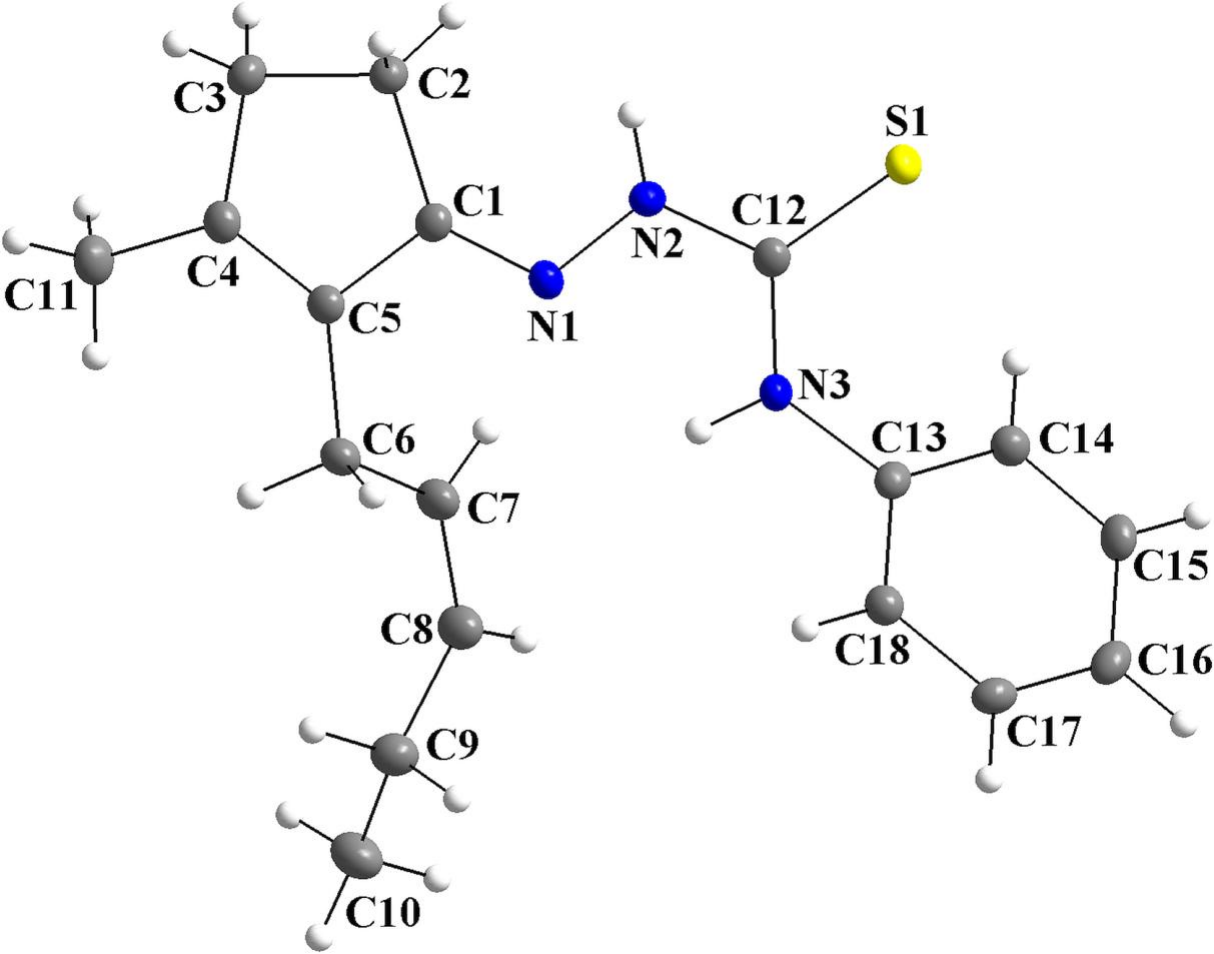
The mol­ecular structure of the title compound, showing the atom labeling and displacement ellipsoids drawn at the 40% probability level.

**Figure 2:**
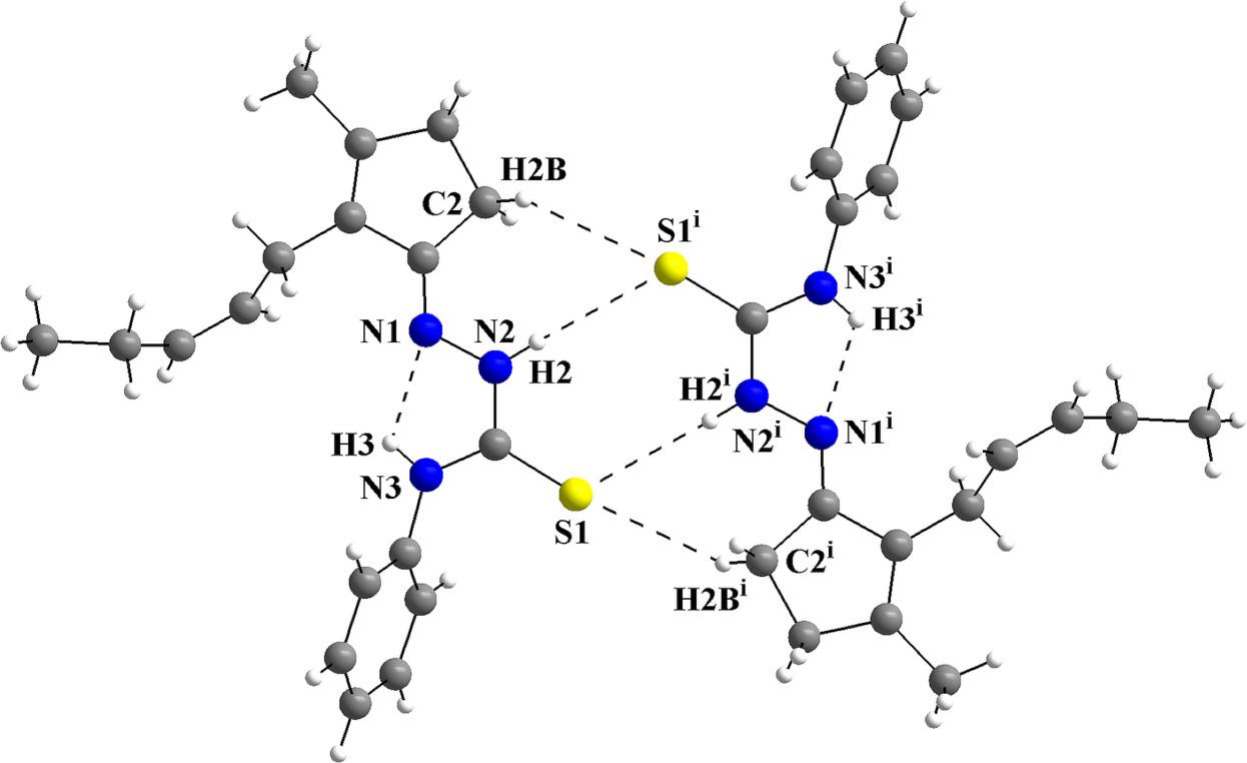
The mol­ecular structure of the *cis*-jasmone 4-phenyl­thio­semicarbazone showing the intra- and inter­molecular hydrogen-bond inter­actions as dashed lines. The mol­ecules are linked into centrosymmetric dimers *via* pairs of N—H⋯S and C—H⋯S inter­actions, forming graph-set motifs 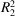 (8) and 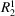 (7). The N—H⋯N intra­molecular inter­actions form rings with graph-set motif *S*(5). [Symmetry code: (i) −*x* + 1, −*y*, −*z*.]

For the title compound, the mol­ecular structure matches the asymmetric unit, with all atoms being located in general positions (Fig. 1 ▸). The thio­semicarbazone fragment is almost planar, with the maximum deviation from the mean plane through the N1/N2/C12/S1/N3 group being 0.0376 (9) Å for N2 and the r.m.s.d. for the selected atoms amounting to 0.0234 Å. The torsion angles of the N1—N2—C12—S1 and N1—N2—C12—N3 chains amount to 176.3 (1) and −5.2 (2)°. The C1–C5 penta­gonal ring is almost planar, as the maximum deviation from the mean plane through the carbon atoms is 0.0117 (1) Å for C5 and the respective r.m.s.d. amounts to 0.0080 Å. The mol­ecule is not planar because of the dihedral angle between the thio­semicarbazone entity and the phenyl ring, which is 56.1 (5)°, and due to the *sp* ^3^-hybridized carbon atoms, *e.g.*, C6 and C9 in the jasmone fragment. In addition, an N3—H3⋯N1 intra­molecular hydrogen bond is observed (Fig. 2 ▸, Table 1 ▸), with graph-set motif *S*(5), which contributes to stabilize the mol­ecular structure.

**Figure 3:**
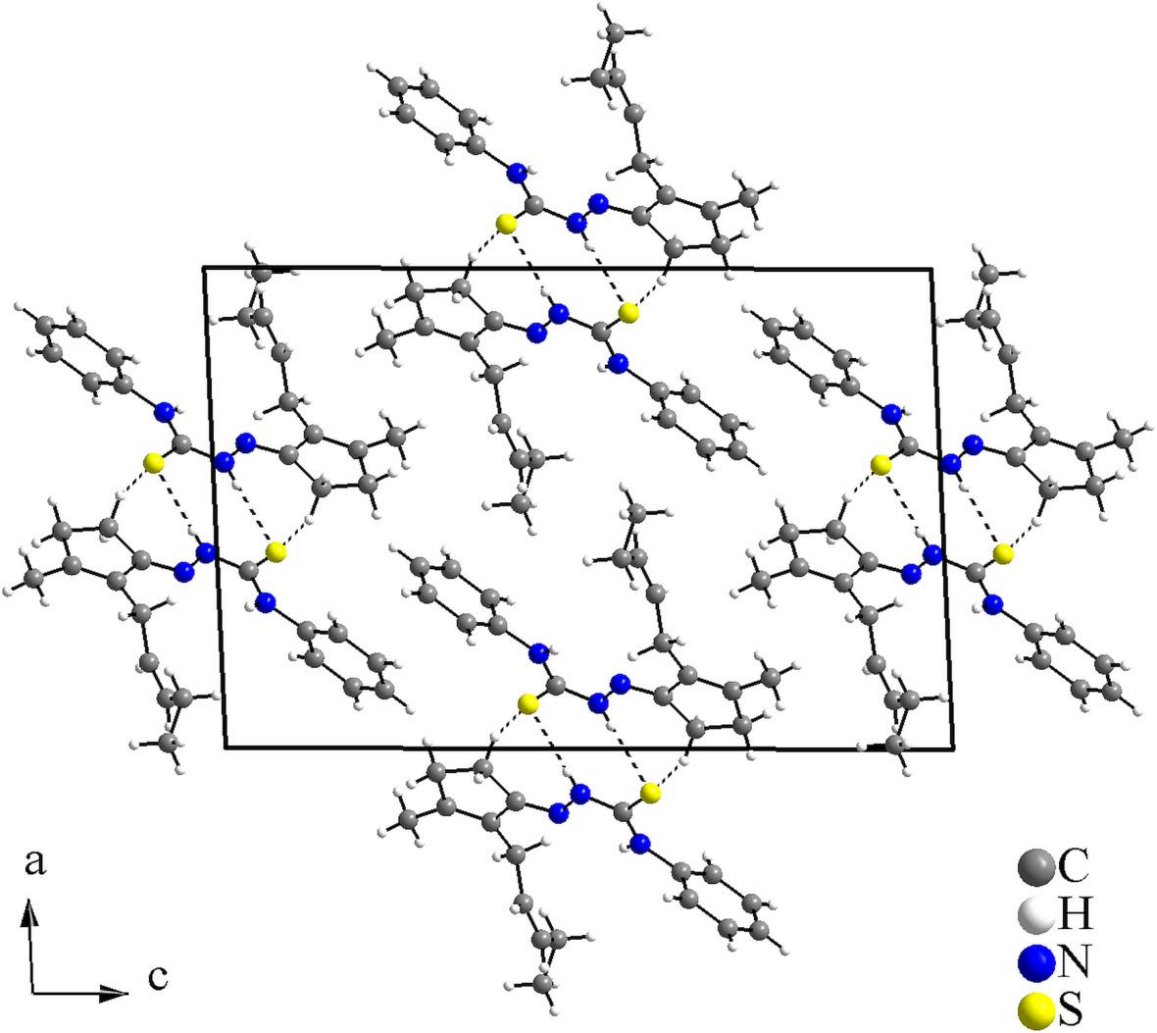
Crystal structure section of the title compound viewed along [010]. The hydrogen-bonding inter­molecular inter­actions are drawn as dashed lines. The crystal structure resembles a zigzag motif when viewed from this direction.

In the crystal, the mol­ecules are connected into centrosymmetric dimers by pairs of N2—H2⋯S1^i^ inter­actions, which form rings of graph-set motif 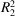 (8) and pairs of N2—H2⋯S1^i^/C2—H2*B*⋯S1^i^ inter­actions, where rings of graph-set motif 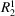 (7) are observed (Fig. 2 ▸, Table 1 ▸). As a feature of the dimeric structure, the sulfur atoms act as double acceptors and three rings with inter­molecular hydrogen bonding are observed. No other strong inter­molecular inter­actions can be suggested for the title compound due to the non-polar organic periphery and the steric effects of the phenyl ring and of the *cis*-jasmone fragment. Only weak inter­actions, *i.e.*, London dispersion forces, can be proposed. The crystal packing resembles a zigzag motif when viewed along [010] (Fig. 3 ▸).

**Figure 4:**
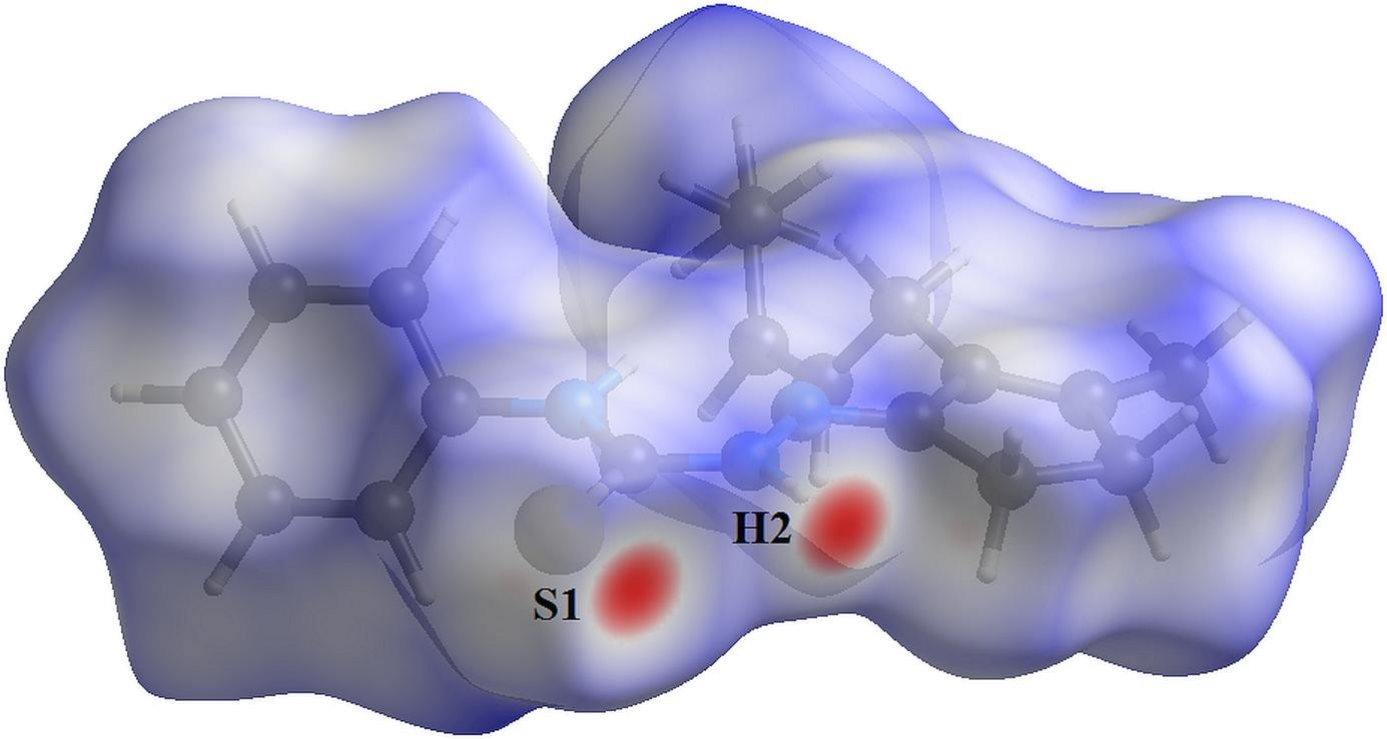
Hirshfeld surface graphical representation (*d*_norm_) for the title compound. The mol­ecule is drawn using a ball-and-stick model, the surface is drawn with transparency and the regions with strongest inter­molecular inter­actions are shown in red and labeled. The figure is simplified for clarity. [*d*_norm_ range: −0.227 to 1.380.]

**Figure 5:**
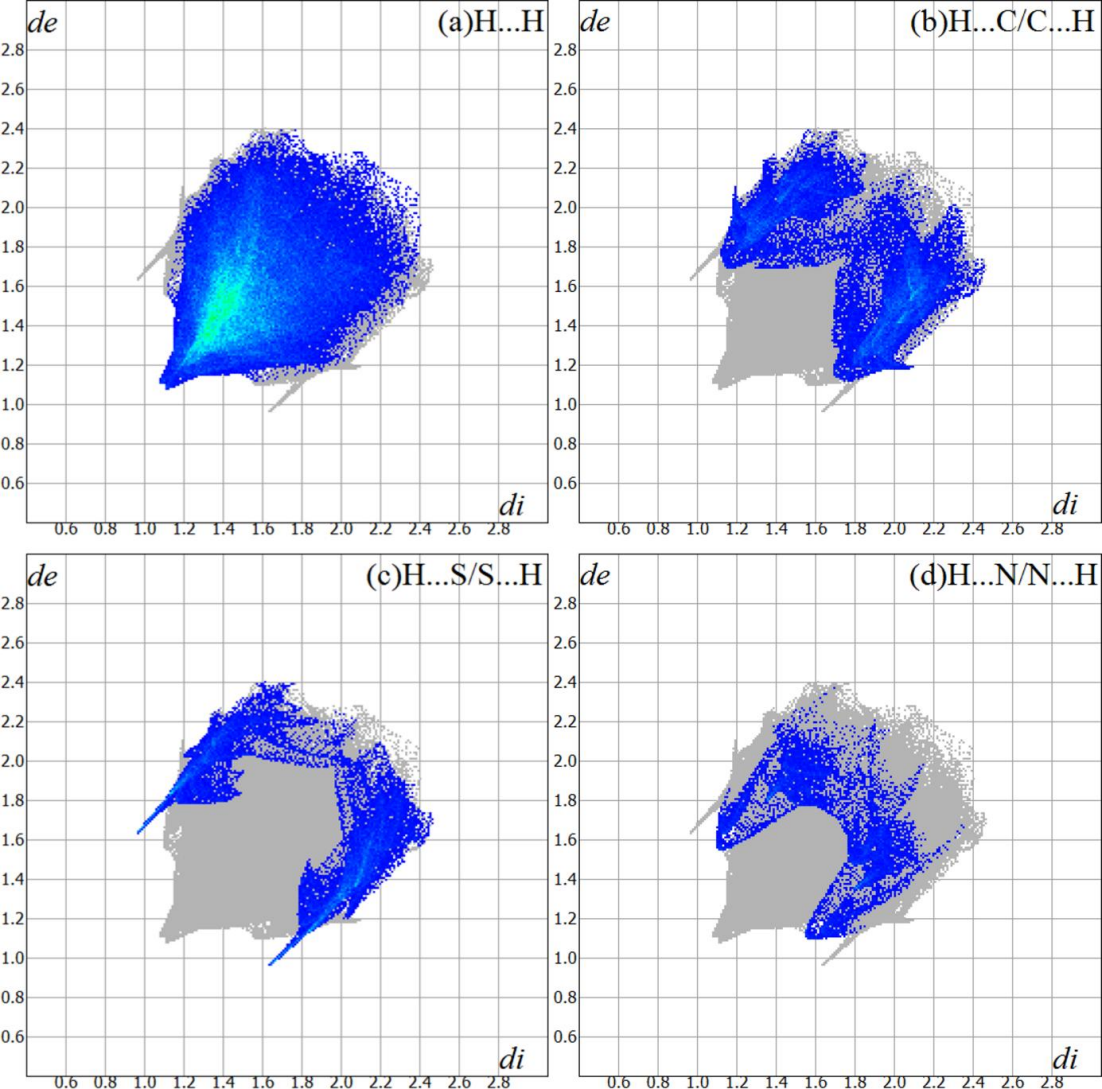
The Hirshfeld surface two-dimensional fingerprint plot (HSFP) for the title compound showing the inter­molecular contacts in detail (cyan dots). The major contributions to the crystal cohesion amount to (*a*) H⋯H = 65.3%, (*b*) H⋯C/C⋯H = 16.2%, (*c*) H⋯S/S⋯H = 10.9% and (*d*) H ⋯N/N⋯H = 5.5%. The *d_i_* (*x*-axis) and the *d_e_* (*y*-axis) values are the closest inter­nal and external distances from given points on the Hirshfeld surface (in Å).

For the title compound, the Hirshfeld surface analysis (Hirshfeld, 1977 ▸), the graphical representations and the two-dimensional Hirshfeld surface fingerprint (HSFP) were evaluated with the *Crystal Explorer* software (Wolff *et al.*, 2012 ▸). The graphical representation of the Hirshfeld surface (*d*_norm_) is represented using a ball-and-stick model with transparency. In red, the locations of the strongest inter­molecular contacts, *i.e*, the regions around the H2 and S1 atoms (Fig. 4 ▸) are indicated. These atoms are those involved in the H⋯S inter­actions showed in the previous figures (Figs. 2 ▸ and 3 ▸). The contributions to the crystal packing are shown as two-dimensional Hirshfeld surface fingerprint plots (HSFP) with cyan dots. The *d_i_* (*x-*axis) and the *d_e_* (*y-*axis) values are the closest inter­nal and external distances from given points on the Hirshfeld surface contacts (in Å). The major contributions to the crystal packing amount to (*a*) H⋯H = 65.3%, (*b*) H⋯C/C⋯H = 16.2%, (*c*) H⋯S/S⋯H = 10.9% and (*d*) H ⋯N/N⋯H = 5.5% (Fig. 5 ▸).

**Figure 6:**
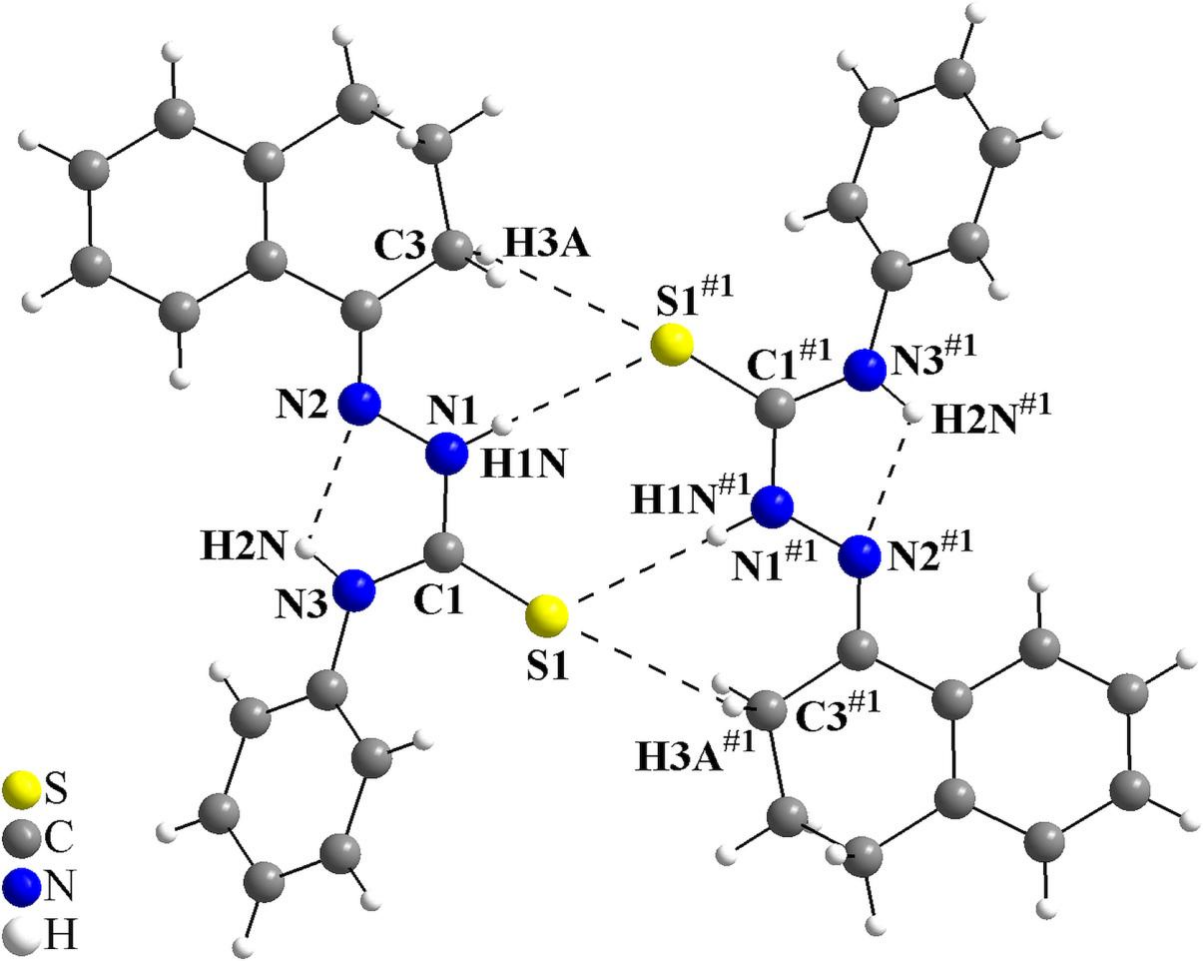
The mol­ecular structure of the reference compound, tetra­lone 4-phenyl­thio­semicarbazone (Oliveira *et al.*, 2014 ▸), showing the intra- and inter­molecular hydrogen-bond inter­actions drawn as dashed lines, which are quite similar to the title compound (Fig. 2 ▸). The mol­ecules are linked into centrosymmetric dimers *via* pairs of N—H⋯S and C—H⋯S inter­actions, forming graph-set motifs of 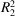 (8) and 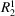 (7). The N—H⋯N intra­molecular inter­actions, which form rings with graph-set motif *S*(5), are also observed. [Symmetry code: (#1) −*x* + 1, −*y*, −*z* + 1.]

**Figure 7:**
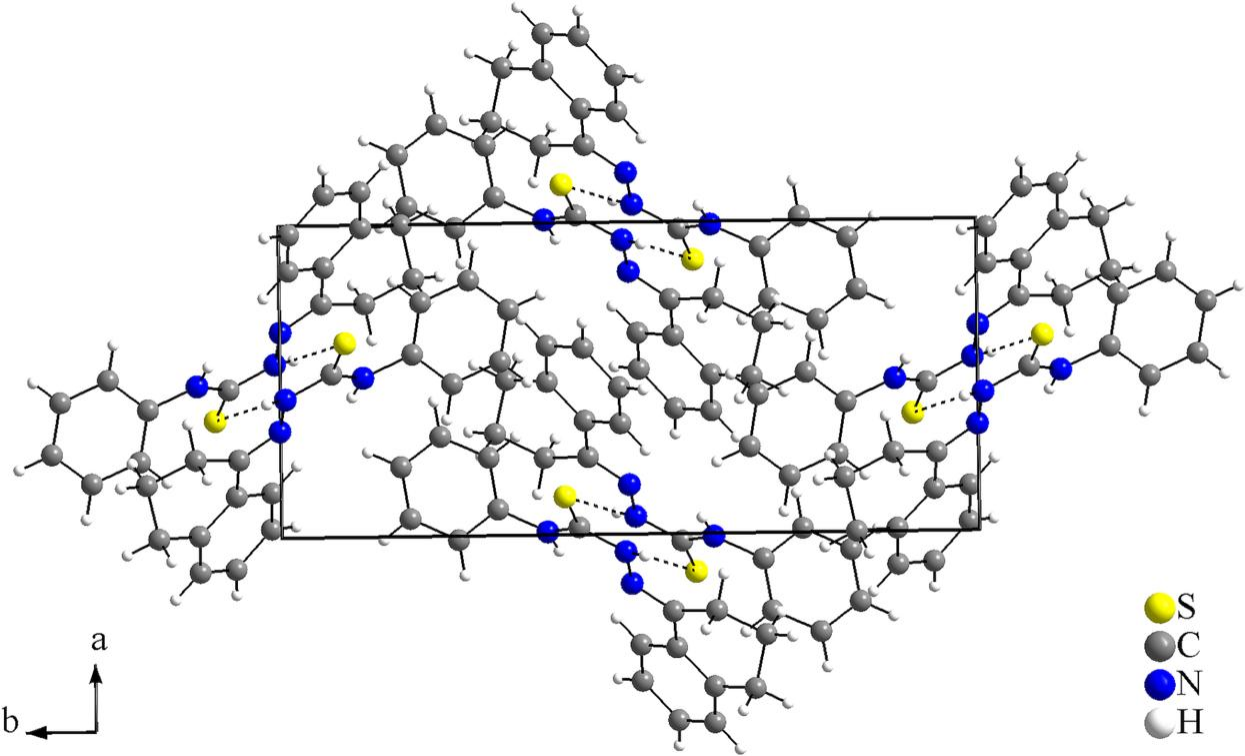
Crystal structure section of the comparison compound, tetra­lone 4-phenyl­thio­semicarbazone (Oliveira *et al.*, 2014 ▸), viewed along [001]. For this view, a zigzag motif of the discrete dimeric units can be suggested. It resembles the packing structure of the title compound (Fig. 3 ▸). Only the inter­molecular N—H⋯S inter­actions are shown for clarity, drawn as dashed lines.

To the best of our knowledge and from using database tools such as *SciFinder* (Chemical Abstracts Service, 2023 ▸) and the Cambridge Structural Database (CSD; Groom *et al.*, 2016 ▸), only the crystal structure of the non-substituted *cis*-jasmone thio­semicarbazone has been reported (Orsoni *et al.*, 2020 ▸). The terminal group of the thio­semicarbazones plays an essential role in the inter­molecular inter­actions and the supra­molecular arrangement, *e.g.*, the non-substituted form, which shows the NH_2_ terminal group, leads to the building of mono-periodic hydrogen-bonded ribbons, while a phenyl ring attached to the terminal nitro­gen atom leads to the formation of discrete dimeric units (Oliveira *et al.*, 2017 ▸). This mol­ecular architecture is specially observed for compounds with a non-polar organic periphery and therefore, the tetra­lone 4-phenyl­thio­semicarbazone derivative (Oliveira *et al.*, 2014 ▸) was chosen for comparison with the title compound. As for the structure of the *cis*-jasmone 4-phenyl­thio­semi­carbazone, an N3—H2*N*⋯N2 intra­molecular inter­action is observed, with graph-set motif *S*(5), and the thio­semicarbazone mol­ecules are linked into centrosymmetric dimers *via* pairs of N1—H1*N*⋯S1^#1^ and C3—H3*A*⋯S1^#1^ inter­actions, forming hydrogen-bonded rings with graph-set motifs of 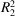 (8) and 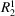 (7). The sulfur atoms also act as double acceptors and, indeed, the intra and inter­molecular hydrogen bonding in the structure of the tetra­lone 4-phenyl­thio­semicarbazone are quite similar to those of the title compound (for the dimeric structure and the symmetry code, see Fig. 6 ▸; for a structural comparison with the compound of this work, see: Fig. 2 ▸). In the crystal, viewed along [001], the tetra­lone 4-phenyl­thio­semicarbazone shows a also zigzag motif, resembling the packing structure of the title compound (Fig. 7 ▸).

## Synthesis and crystallization

The starting materials are commercially available and were used without further purification. The synthesis was adapted from previously reported procedures (Freund & Schander, 1902 ▸; Oliveira *et al.*, 2014 ▸). The hydro­chloric acid-catalyzed reaction between *cis*-jasmone (8 mmol) and 4-phenyl­thio­semicarbazide (8 mmol) in ethanol (80 ml) was refluxed for 6 h. After cooling and filtering, the title compound was obtained as precipitate, filtered off and washed with cold ethanol. Colorless single crystals suitable for X-ray diffraction were obtained in tetra­hydro­furan by slow evaporation of the solvent.

## Refinement

Crystal data, data collection and structure refinement details are summarized in Table 2 ▸.

## Supplementary Material

Crystal structure: contains datablock(s) I. DOI: 10.1107/S2414314623009719/bt4142sup1.cif

Structure factors: contains datablock(s) I. DOI: 10.1107/S2414314623009719/bt4142Isup2.hkl

Supporting information file. DOI: 10.1107/S2414314623009719/bt4142Isup3.cml

CCDC reference: 2304274

Additional supporting information:  crystallographic information; 3D view; checkCIF report

## Figures and Tables

**Table 1:** Hydrogen-bond geometry (Å, °)

*D*—H⋯*A*	*D*—H	H⋯*A*	*D*⋯*A*	*D*—H⋯*A*
C2—H2*B*⋯S1^i^	0.97	2.96	3.4640 (16)	113
N2—H2⋯S1^i^	0.86	2.72	3.5757 (13)	177
N3—H3⋯N1	0.86	2.11	2.5457 (18)	111

Symmetry code: (i) 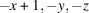 .

**Table 2:** Experimental details

Crystal data	
Chemical formula	C_18_H_23_N_3_S
*M*_r_	313.45
Crystal system, space group	Monoclinic, *P*2_1_/*n*
Temperature (K)	123
*a*, *b*, *c* (Å)	13.6565 (3), 5.8286 (2), 20.6721 (6)
β (°)	92.751 (2)
*V* (Å^3^)	1643.57 (8)
*Z*	4
Radiation type	Mo *K*α
μ (mm^−1^)	0.20
Crystal size (mm)	0.22 × 0.13 × 0.05

Data collection	
Diffractometer	Enraf–Nonius FR590 Kappa CCD
No. of measured, independent and observed [*I* > 2σ(*I*)] reflections	26959, 3751, 2857
*R*_int_	0.064
(sin θ/λ)_max_ (Å^−1^)	0.649

Refinement	
*R*[*F* ^2^ > 2σ(*F* ^2^)], *wR*(*F* ^2^), *S*	0.039, 0.095, 1.06
No. of reflections	3751
No. of parameters	202
H-atom treatment	H-atom parameters constrained
Δρ_max_, Δρ_min_ (e Å^−3^)	0.28, −0.25

Computer programs: *COLLECT* (Nonius, 1998 ▸), *HKL* *DENZO* and *SCALEPACK* (Otwinowski & Minor, 1997 ▸), *SIR92* (Altomare *et al.*, 1999 ▸), *SHELXL2018/3* (Sheldrick, 2015 ▸), *DIAMOND* (Brandenburg, 2006 ▸), *CrystalExplorer* (Wolff *et al.*, 2012 ▸), *WinGX* (Farrugia, 2012 ▸), *publCIF* (Westrip, 2010 ▸) and *enCIFer* (Allen *et al.*, 2004 ▸).

## full crystallographic data

### Crystal data

**Table:**

C_18_H_23_N_3_S	*F*(000) = 672
*M**_r_* = 313.45	*D*_x_ = 1.267 Mg m^−^^3^
Monoclinic, *P*2_1_/*n*	Mo *K*α radiation, λ = 0.71073 Å
*a* = 13.6565 (3) Å	Cell parameters from 63134 reflections
*b* = 5.8286 (2) Å	θ = 2.9–27.5°
*c* = 20.6721 (6) Å	µ = 0.20 mm^−^^1^
β = 92.751 (2)°	*T* = 123 K
*V* = 1643.57 (8) Å^3^	Fragment, colourless
*Z* = 4	0.22 × 0.13 × 0.05 mm

### Data collection

**Table:**

Enraf–Nonius FR590 Kappa CCD diffractometer	2857 reflections with *I* > 2σ(*I*)
Radiation source: sealed X-ray tube, Enraf–Nonius FR590	*R*_int_ = 0.064
Detector resolution: 9 pixels mm^-1^	θ_max_ = 27.5°, θ_min_ = 3.0°
CCD rotation images, thick slices, κ–goniostat scans	*h* = −17→17
26959 measured reflections	*k* = −7→7
3751 independent reflections	*l* = −26→26

### Refinement

**Table:**

Refinement on *F*^2^	Hydrogen site location: inferred from neighbouring sites
Least-squares matrix: full	H-atom parameters constrained
*R*[*F*^2^ > 2σ(*F*^2^)] = 0.039	*w* = 1/[σ^2^(*F*_o_^2^) + (0.0359*P*)^2^ + 0.7368*P*] where *P* = (*F*_o_^2^ + 2*F*_c_^2^)/3
*wR*(*F*^2^) = 0.095	(Δ/σ)_max_ < 0.001
*S* = 1.06	Δρ_max_ = 0.28 e Å^−^^3^
3751 reflections	Δρ_min_ = −0.25 e Å^−^^3^
202 parameters	Extinction correction: SHELXL-2018/3 (Sheldrick 2015), Fc^*^=kFc[1+0.001xFc^2^λ^3^/sin(2θ)]^-1/4^
0 restraints	Extinction coefficient: 0.0053 (9)
Primary atom site location: structure-invariant direct methods	

### Special details

**Table:**

Geometry. All esds (except the esd in the dihedral angle between two l.s. planes) are estimated using the full covariance matrix. The cell esds are taken into account individually in the estimation of esds in distances, angles and torsion angles; correlations between esds in cell parameters are only used when they are defined by crystal symmetry. An approximate (isotropic) treatment of cell esds is used for estimating esds involving l.s. planes.
Refinement. An absorption correction was not performed, as the crystal data analysis suggested that the absorption effects were not significant for the structure refinement. Hydrogen atoms were located in a difference-Fourier map, but were positioned with idealized geometry and refined isotropically using a riding model (HFIX command). Methyl H atoms were allowed to rotate but not to tip to best fit the experimental electron density. Thus, for the methyl H atoms [*U*_iso_(H) = 1.5 *U*_eq_(C)], the C—H bond lengths were set to 0.96 Å. The other C—H bond lengths were also set according to the H atom neighbourhood [*U*_iso_(H) = 1.2 *U*_eq_(C)]. For the phenyl H atoms and for the other H atoms attached to *sp*^2^-hybridized carbon atoms (C7 and C8), the C—H bond lengths were set 0.93 Å. For the H atoms of the —CH_2_— fragments (C2, C3, C6 and C9), the C—H bond lengths were set to 0.97 Å. Finally, the N—H bond lengths [*U*_iso_(H) = 1.2 *U*_eq_(N)] were set to 0.86 Å.

### Fractional atomic coordinates and isotropic or equivalent isotropic displacement parameters (Å^2^)

**Table:**

	*x*	*y*	*z*	*U*_iso_*/*U*_eq_	
C1	0.38987 (11)	0.5560 (3)	−0.10461 (7)	0.0182 (3)	
C2	0.45778 (11)	0.4298 (3)	−0.14766 (8)	0.0207 (3)	
H2A	0.436900	0.272027	−0.154050	0.025*	
H2B	0.524485	0.431089	−0.129277	0.025*	
C3	0.45002 (12)	0.5646 (3)	−0.21191 (8)	0.0239 (4)	
H3A	0.513393	0.625665	−0.222395	0.029*	
H3B	0.425872	0.466784	−0.247139	0.029*	
C4	0.37868 (11)	0.7559 (3)	−0.20041 (8)	0.0205 (3)	
C5	0.34692 (11)	0.7526 (3)	−0.13988 (7)	0.0189 (3)	
C6	0.27920 (11)	0.9155 (3)	−0.10790 (8)	0.0220 (3)	
H6A	0.274508	1.055634	−0.133179	0.026*	
H6B	0.307436	0.954690	−0.065378	0.026*	
C7	0.17720 (12)	0.8219 (3)	−0.10048 (8)	0.0255 (4)	
H7	0.165822	0.670153	−0.112577	0.031*	
C8	0.10260 (12)	0.9383 (3)	−0.07815 (9)	0.0277 (4)	
H8	0.043700	0.859385	−0.075799	0.033*	
C9	0.10293 (12)	1.1834 (3)	−0.05628 (9)	0.0275 (4)	
H9A	0.159387	1.260861	−0.072846	0.033*	
H9B	0.109126	1.188208	−0.009357	0.033*	
C10	0.01004 (13)	1.3102 (3)	−0.07920 (10)	0.0337 (4)	
H10A	0.006058	1.315650	−0.125670	0.051*	
H10B	0.011733	1.463645	−0.062278	0.051*	
H10C	−0.046191	1.231343	−0.064102	0.051*	
C11	0.35163 (13)	0.9202 (3)	−0.25364 (8)	0.0266 (4)	
H11A	0.304018	1.027255	−0.239068	0.040*	
H11B	0.324443	0.837326	−0.290397	0.040*	
H11C	0.409031	1.001812	−0.265701	0.040*	
C12	0.36853 (11)	0.2620 (3)	0.04217 (7)	0.0182 (3)	
C13	0.24371 (11)	0.3876 (3)	0.11856 (7)	0.0189 (3)	
C14	0.18756 (11)	0.1912 (3)	0.12727 (8)	0.0214 (3)	
H14	0.190844	0.068671	0.098647	0.026*	
C15	0.12685 (11)	0.1804 (3)	0.17895 (8)	0.0235 (4)	
H15	0.088949	0.050178	0.184838	0.028*	
C16	0.12210 (11)	0.3623 (3)	0.22203 (8)	0.0241 (4)	
H16	0.081198	0.353730	0.256649	0.029*	
C17	0.17823 (12)	0.5562 (3)	0.21342 (8)	0.0232 (4)	
H17	0.175481	0.677598	0.242474	0.028*	
C18	0.23886 (11)	0.5702 (3)	0.16133 (8)	0.0215 (3)	
H18	0.276003	0.701443	0.155225	0.026*	
N1	0.36550 (9)	0.5130 (2)	−0.04654 (6)	0.0189 (3)	
N2	0.40538 (9)	0.3219 (2)	−0.01525 (6)	0.0195 (3)	
H2	0.451694	0.244307	−0.031612	0.023*	
N3	0.30211 (9)	0.4113 (2)	0.06367 (6)	0.0201 (3)	
H3	0.294026	0.535658	0.041653	0.024*	
S1	0.40757 (3)	0.02358 (7)	0.08139 (2)	0.02292 (12)	

### Atomic displacement parameters (Å^2^)

**Table:**

	*U*^11^	*U*^22^	*U*^33^	*U*^12^	*U*^13^	*U*^23^
C1	0.0171 (8)	0.0187 (8)	0.0187 (8)	−0.0025 (6)	0.0011 (6)	−0.0009 (6)
C2	0.0207 (8)	0.0204 (8)	0.0210 (8)	0.0008 (6)	0.0031 (6)	0.0008 (6)
C3	0.0267 (9)	0.0242 (8)	0.0212 (8)	0.0006 (7)	0.0063 (7)	0.0007 (7)
C4	0.0193 (8)	0.0204 (8)	0.0216 (8)	−0.0030 (6)	0.0006 (6)	0.0015 (6)
C5	0.0180 (8)	0.0174 (8)	0.0212 (8)	−0.0024 (6)	0.0015 (6)	−0.0003 (6)
C6	0.0231 (8)	0.0177 (8)	0.0254 (9)	0.0008 (6)	0.0037 (7)	−0.0001 (7)
C7	0.0259 (9)	0.0192 (8)	0.0318 (10)	−0.0013 (7)	0.0047 (7)	−0.0009 (7)
C8	0.0236 (9)	0.0238 (9)	0.0361 (10)	−0.0022 (7)	0.0053 (7)	−0.0004 (7)
C9	0.0272 (9)	0.0252 (9)	0.0302 (9)	0.0012 (7)	0.0027 (7)	−0.0026 (7)
C10	0.0309 (10)	0.0251 (9)	0.0452 (12)	0.0030 (7)	0.0022 (8)	0.0007 (8)
C11	0.0294 (9)	0.0271 (9)	0.0231 (9)	−0.0017 (7)	−0.0006 (7)	0.0048 (7)
C12	0.0192 (8)	0.0180 (8)	0.0174 (8)	−0.0028 (6)	−0.0003 (6)	−0.0013 (6)
C13	0.0180 (8)	0.0207 (8)	0.0181 (8)	0.0030 (6)	0.0019 (6)	0.0030 (6)
C14	0.0234 (8)	0.0204 (8)	0.0205 (8)	−0.0004 (6)	0.0010 (7)	0.0003 (6)
C15	0.0213 (8)	0.0239 (8)	0.0255 (9)	−0.0034 (7)	0.0032 (7)	0.0051 (7)
C16	0.0203 (8)	0.0317 (9)	0.0207 (8)	0.0034 (7)	0.0042 (7)	0.0043 (7)
C17	0.0239 (8)	0.0244 (9)	0.0216 (8)	0.0048 (7)	0.0026 (7)	−0.0026 (6)
C18	0.0214 (8)	0.0199 (8)	0.0234 (8)	−0.0004 (6)	0.0027 (6)	0.0010 (6)
N1	0.0213 (7)	0.0165 (6)	0.0189 (7)	−0.0007 (5)	0.0010 (5)	0.0019 (5)
N2	0.0216 (7)	0.0183 (6)	0.0188 (7)	0.0029 (5)	0.0034 (5)	0.0011 (5)
N3	0.0240 (7)	0.0171 (6)	0.0195 (7)	0.0018 (5)	0.0057 (5)	0.0036 (5)
S1	0.0255 (2)	0.0203 (2)	0.0233 (2)	0.00340 (16)	0.00521 (16)	0.00501 (16)

### Geometric parameters (Å, º)

**Table:**

C1—N1	1.286 (2)	C10—H10B	0.9600
C1—C5	1.466 (2)	C10—H10C	0.9600
C1—C2	1.507 (2)	C11—H11A	0.9600
C2—C3	1.542 (2)	C11—H11B	0.9600
C2—H2A	0.9700	C11—H11C	0.9600
C2—H2B	0.9700	C12—N3	1.348 (2)
C3—C4	1.507 (2)	C12—N2	1.357 (2)
C3—H3A	0.9700	C12—S1	1.6827 (16)
C3—H3B	0.9700	C13—C18	1.387 (2)
C4—C5	1.344 (2)	C13—C14	1.394 (2)
C4—C11	1.492 (2)	C13—N3	1.425 (2)
C5—C6	1.501 (2)	C14—C15	1.385 (2)
C6—C7	1.511 (2)	C14—H14	0.9300
C6—H6A	0.9700	C15—C16	1.388 (2)
C6—H6B	0.9700	C15—H15	0.9300
C7—C8	1.325 (2)	C16—C17	1.382 (2)
C7—H7	0.9300	C16—H16	0.9300
C8—C9	1.498 (2)	C17—C18	1.392 (2)
C8—H8	0.9300	C17—H17	0.9300
C9—C10	1.524 (2)	C18—H18	0.9300
C9—H9A	0.9700	N1—N2	1.3863 (18)
C9—H9B	0.9700	N2—H2	0.8600
C10—H10A	0.9600	N3—H3	0.8600
			
N1—C1—C5	120.09 (14)	C9—C10—H10B	109.5
N1—C1—C2	130.57 (14)	H10A—C10—H10B	109.5
C5—C1—C2	109.32 (13)	C9—C10—H10C	109.5
C1—C2—C3	103.95 (12)	H10A—C10—H10C	109.5
C1—C2—H2A	111.0	H10B—C10—H10C	109.5
C3—C2—H2A	111.0	C4—C11—H11A	109.5
C1—C2—H2B	111.0	C4—C11—H11B	109.5
C3—C2—H2B	111.0	H11A—C11—H11B	109.5
H2A—C2—H2B	109.0	C4—C11—H11C	109.5
C4—C3—C2	105.03 (13)	H11A—C11—H11C	109.5
C4—C3—H3A	110.7	H11B—C11—H11C	109.5
C2—C3—H3A	110.7	N3—C12—N2	113.96 (13)
C4—C3—H3B	110.7	N3—C12—S1	125.25 (12)
C2—C3—H3B	110.7	N2—C12—S1	120.78 (12)
H3A—C3—H3B	108.8	C18—C13—C14	120.27 (14)
C5—C4—C11	128.03 (15)	C18—C13—N3	118.60 (14)
C5—C4—C3	112.11 (14)	C14—C13—N3	121.01 (14)
C11—C4—C3	119.85 (14)	C15—C14—C13	119.37 (15)
C4—C5—C1	109.55 (14)	C15—C14—H14	120.3
C4—C5—C6	129.38 (15)	C13—C14—H14	120.3
C1—C5—C6	121.06 (14)	C14—C15—C16	120.55 (15)
C5—C6—C7	114.00 (13)	C14—C15—H15	119.7
C5—C6—H6A	108.8	C16—C15—H15	119.7
C7—C6—H6A	108.8	C17—C16—C15	119.91 (15)
C5—C6—H6B	108.8	C17—C16—H16	120.0
C7—C6—H6B	108.8	C15—C16—H16	120.0
H6A—C6—H6B	107.6	C16—C17—C18	120.12 (15)
C8—C7—C6	125.32 (15)	C16—C17—H17	119.9
C8—C7—H7	117.3	C18—C17—H17	119.9
C6—C7—H7	117.3	C13—C18—C17	119.78 (15)
C7—C8—C9	127.02 (16)	C13—C18—H18	120.1
C7—C8—H8	116.5	C17—C18—H18	120.1
C9—C8—H8	116.5	C1—N1—N2	118.59 (13)
C8—C9—C10	112.23 (15)	C12—N2—N1	117.45 (12)
C8—C9—H9A	109.2	C12—N2—H2	121.3
C10—C9—H9A	109.2	N1—N2—H2	121.3
C8—C9—H9B	109.2	C12—N3—C13	127.68 (13)
C10—C9—H9B	109.2	C12—N3—H3	116.2
H9A—C9—H9B	107.9	C13—N3—H3	116.2
C9—C10—H10A	109.5		
			
N1—C1—C2—C3	177.17 (16)	C18—C13—C14—C15	0.1 (2)
C5—C1—C2—C3	−1.15 (17)	N3—C13—C14—C15	−175.86 (14)
C1—C2—C3—C4	0.01 (16)	C13—C14—C15—C16	−0.4 (2)
C2—C3—C4—C5	1.26 (18)	C14—C15—C16—C17	0.1 (2)
C2—C3—C4—C11	−178.74 (14)	C15—C16—C17—C18	0.5 (2)
C11—C4—C5—C1	177.96 (15)	C14—C13—C18—C17	0.5 (2)
C3—C4—C5—C1	−2.03 (18)	N3—C13—C18—C17	176.55 (14)
C11—C4—C5—C6	−2.6 (3)	C16—C17—C18—C13	−0.8 (2)
C3—C4—C5—C6	177.45 (15)	C5—C1—N1—N2	178.18 (13)
N1—C1—C5—C4	−176.51 (14)	C2—C1—N1—N2	0.0 (2)
C2—C1—C5—C4	2.01 (18)	N3—C12—N2—N1	−5.15 (19)
N1—C1—C5—C6	4.0 (2)	S1—C12—N2—N1	176.26 (10)
C2—C1—C5—C6	−177.53 (13)	C1—N1—N2—C12	−171.07 (14)
C4—C5—C6—C7	104.92 (19)	N2—C12—N3—C13	173.62 (14)
C1—C5—C6—C7	−75.65 (19)	S1—C12—N3—C13	−7.9 (2)
C5—C6—C7—C8	−174.86 (17)	C18—C13—N3—C12	132.16 (16)
C6—C7—C8—C9	0.4 (3)	C14—C13—N3—C12	−51.8 (2)
C7—C8—C9—C10	138.6 (2)		

### Hydrogen-bond geometry (Å, º)

**Table:**

*D*—H···*A*	*D*—H	H···*A*	*D*···*A*	*D*—H···*A*
C2—H2*B*···S1^i^	0.97	2.96	3.4640 (16)	113
N2—H2···S1^i^	0.86	2.72	3.5757 (13)	177
N3—H3···N1	0.86	2.11	2.5457 (18)	111

Symmetry code: (i) −*x*+1, −*y*, −*z*.
