# 2-{3-Methyl-2-[(2*Z*)-pent-2-en-1-yl]cyclo­pent-2-en-1-yl­idene}-*N*-phenylhydrazinecarbo­thio­amide. Corrigendum

**DOI:** 10.1107/S2414314624000099

**Published:** 2024-01-31

**Authors:** Adriano Bof de Oliveira, Leandro Bresolin, Johannes Beck, Jörg Daniels

**Affiliations:** a Departamento de Química, Universidade Federal de Sergipe, Av. Marcelo Deda Chagas s/n, Campus Universitário, 49107-230 São Cristóvão-SE, Brazil; b Escola de Química e Alimentos, Universidade Federal do Rio Grande, Av. Itália km 08, Campus Carreiros, 96203-900 Rio Grande-RS, Brazil; c Institut für Anorganische Chemie, Rheinische Friedrich-Wilhelms-Universität Bonn, Gerhard-Domagk-Strasse 1, D-53121 Bonn, Germany

## Abstract

In the paper by Oliveira *et al.* [*IUCrData* (2023), **8**, x230971], there was an error in the name of the first author.

## Abstract 2

Corrigendum to *IUCrData* (2023), **8**, x230971.

The name of the first author in the paper by Oliveira *et al.* (2023 ▸) is incorrect and should be ‘Adriano Bof de Oliveira’, as given above.
